# Antioxidant Activity of New Copolymer Conjugates of Methoxyoligo(Ethylene Glycol)Methacrylate and Betulin Methacrylate with Cerium Oxide Nanoparticles *In Vitro*

**DOI:** 10.3390/molecules27185894

**Published:** 2022-09-11

**Authors:** Nina Melnikova, Dmitry Orekhov, Alexander Simagin, Darina Malygina, Vitaly Korokin, Karina Kosmachova, Haider Al-Azzavi, Anna Solovyeva, Oleg Kazantsev

**Affiliations:** 1 Faculty of Chemistry, Lobachevsky University, 23/5 Gagarin Av., 603950 Nizhny Novgorod, Russia; 2 Research Laboratory “New Polymeric Materials”, Nizhny Novgorod State Technical University n.a. R.E. Alekseev, 24 Minin St., 603950 Nizhny Novgorod, Russia; 3 Department of Pharmaceutical Chemistry, Privolzhsky Research Medical University, 10/1 Minin Sq., 603950 Nizhny Novgorod, Russia; 4 Central Research Laboratory, Privolzhsky Research Medical University, 10/1 Minin Sq., 603950 Nizhny Novgorod, Russia

**Keywords:** betulin, copolymer of methoxyoligo(ethylene glycol)methacrylate, betulin methacrylate, cerium oxide nanoparticles, combination therapy, antioxidant properties

## Abstract

The synthesis of two new copolymer conjugates of methoxyoligo(ethylene glycol)methacrylate MPEGMA and betulin methacrylate BM was developed via RAFT polymerization. The molar content of BM units was equal to 9–10 and 13–16 mol%, respectively (HPLC, ^1^H and ^13^C NMR); molar weights were equal to 75000–115000. CeO_2_ NPs as a component of the hybrid material were synthesized for the preparation of the composition with copolymer conjugates of MPEGMA and BM. We showed a significant increase in G6PDH and GR activities by 21–51% and 9–132%, respectively, which was due to the increase in NADPH concentration under the action of copolymers *in vitro*. The actions of copolymers and CeO_2_ NPs combination were stronger than those of the individual components: the SOD activity increased by more than 30%, the catalase activity increased dose-dependently from 13 to 45%, and the GR activity increased to 49%. The maximum increase in enzyme activity was observed for the G6PDH from 54% to 151%. The MDA level dose-dependently increased by 3–15% under the action of copolymers compared with the control, and dose-dependently decreased by 3–12% in samples containing CeO_2_ NPs only. CeO_2_ NP–copolymer compositions can be used for the design of new biomimetic medical products with controlled antioxidant properties.

## 1. Introduction

Drugs and biologically active compounds that control antioxidant and pro-oxidant properties are very important for the treatment of diseases, such as systemic inflammatory response syndrome, critical limb ischemia, diabetes mellitus, age-related muscular degeneration, and cancer, that have oxidative stress as a major trigger. Betulin is a naturally occurring triterpene of the white birch bark and shows anticancer, antiviral, hepatoprotective, and other activities due to a balance of antioxidant and pro-oxidant properties [1,2,3,4,5]. Betulin induces reactive oxygen species (ROS)-dependant apoptosis in human gastric cancer [6] and other types of cancer [3]. The generation of ROS is suppressed by betulin treatment in a time- and dose-dependent manner by preventing tyrosyl phosphorylation in human neutrophils [7]. Betulin promotes the normalization of the catalase level, reduced glutathione content, and the activities of glutathione peroxidase and glutathione reductase, which are the key enzymes of the reduced glutathione cycle in erythrocytes [8]. Betulin demonstrated a protective effect on chronic obstructive pulmonary disease via antioxidant properties due to restoration of the activities of superoxide dismutase (SOD) in serum and lungs and catalase in serum, and a reduction in the content of malonic dialdehyde (MDA) [9]. Betulin also inhibits the overproduction of pro-inflammatory cytokines, including tumor necrosis factor α, interleukin-6, and interleukin-1β [9].

The major problem limiting their potential pharmaceutical uses is the poor aqueous solubility of betulin when trying to obtain a pharmaceutical formulation with an easily administrated betulin release system.

Amphiphilic copolymers with strong biological activity and low toxicity may be used as an interesting building block for betulin and its derivatives for solubility improvement. The conjugates of betulinic acid with PEGs and other kinds of polymers enhance the anticancer potential compared with native betulinic acid in vivo [10,11,12]. The micellar polymer–betulinic acid conjugates based on N-(2-hydroxypropyl)-methacrylamide copolymer carriers enabled the controlled release of cytotoxic BA derivatives in solid tumor or tumor cells [13].

The betulin bioavailability improvement was achieved via the synthesis of conjugates of poly(N-isopropylacrylamide-co-acrylic acid) and poly(N-vinylpyrrolidone-co-acrylic acid) copolymers with betulin and water-soluble bovine serum albumin (BSA)–betulin covalent conjugates for cancer treatment [14]. More simple polymers with acrylated betulin as a comonomer may be used for the design of bio-based resins for coating [15]. The synthesis of a betulin-based polyanhydride that exhibits anticancer effects was described by D. Niewolic [16]. A polymer based on a copolymer of N-vinylpyrrolidone with N-(n-carboxy)-phenylmaleimide and betulin has a higher activity against melanoma compared with betulin [17]. Methacrylate-functionalized betulin derivatives as antibacterial comonomer were proposed as dental restorative resins [18]. Glycopolymers bearing galactose and betulin with methacrylate as monomers using RAFT-polymerization may serve as biocompatible multifunctional biomaterials and carriers for use in the targeted release of drugs [19]. Thus, a polymer-conjugated betulin system (for example, as micelles) may be used not only as the betulin carrier but also allows for including other active pharmaceutical ingredients.

Ceria (CeO_2_ NPs) nanostructures also have antioxidant or pro-oxidant properties depending on the experimental condition and ceria nanomorphology [20,21,22,23,24,25,26,27,28,29,30,31,32,33,34]. Ceria exists in a trivalent state (^+3^) or a tetravalent state (^+4^) and may change between the two in a redox reaction. It was established that cerium oxide makes an excellent oxygen buffer because of this redox capacity. It exhibits versatile catalytic properties, including multi-enzyme-mimetic properties. Ceria nanoparticles demonstrate a synergetic effect with well-known anticancer drugs or other therapeutic ingredients, enhancing their effects [20,21,22,23,24,25,26,27,28,29,30,31,32,33,34]. A combination of classical chemotherapeutic agents with non-genotoxic but active antitumor CeO_2_ NPs may provide a new strategy against diseases caused by oxidative stress [29].

In this study, we developed the synthesis of new antioxidant–copolymer conjugates of methoxyoligo(ethylene glycol)methacrylate and betulin methacrylate and studied the antioxidant and pro-oxidant effects of new copolymer conjugates in combination with ceria nanoparticles *in vitro*.

## 2. Results

### 2.1. Polymer Properties

**Figure 1:**
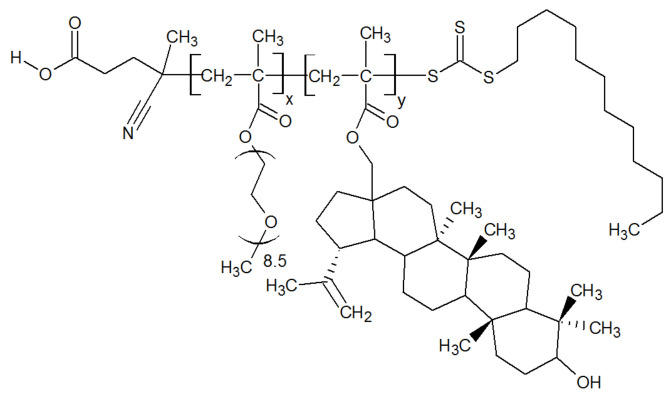
General structural formula of synthesized copolymers of methoxyoligo(ethylene glycol)methacrylate and betulin methacrylate.

Figure 1 shows the general structural formula of the newly synthesized copolymers of methoxyoligo(ethylene glycol)methacrylate and betulin methacrylate. Two independent methods were used to determine the copolymer compositions. Using the first method, the average copolymer composition was found in situ as the difference between the initial and final concentrations of comonomers in the polymerization mixtures, i.e., according to the ratios of the number of reacted comonomers. It was determined using HPLC. Typical chromatograms are shown in Figure S1 and Table S1. The molar contents of betulin methacrylate units determined using this method were equal to 9.3 mol% and 13.3 mol% for Bet-1 and Bet-2, consequently.

**Figure 2:**
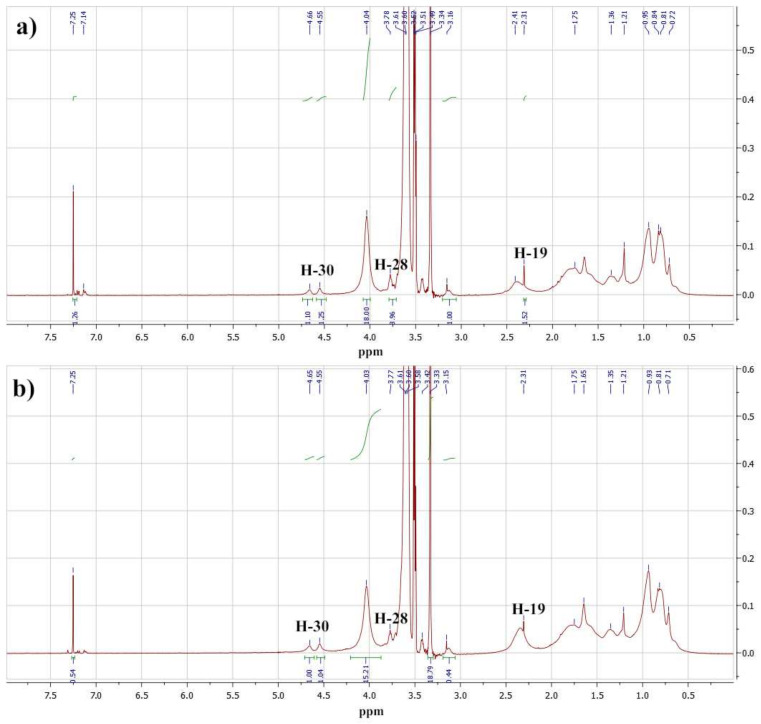
Fragments of the ^1^H NMR spectra of the Bet-1 (**a**) and Bet-2 (**b**) copolymers.

From the second method, the copolymer composition of the final isolated products was determined using ^1^H NMR spectra. Figure 2 shows fragments of the ^1^H NMR spectra of the Bet-1 and Bet-2 copolymers. These spectra were compared with the spectrum of the initial betulin from the Betulin Project NMR Spectra (2014) [35]. The signals of the protons of the main chain and side betulin (4.56 and 4.67 ppm of the olefin protons H-30 of betulin) and oligoethylene glycol fragments were identified in this region of the copolymer spectra. Broad signals in the region of 0.7–2.0 ppm that corresponded to the hydrogen atoms of the lupane fragment and the broad singlet at 4.04 ppm were due to the proton of the hydroxyl group at C-3 of the betulin backbone. Signals of the C-12 alkyl fragment appeared in the same range of the spectrum. Signals at 4.04 and 3.34 ppm corresponded to the methylene fragment at the carboxyl group and the terminal methyl group of the oligoethylene glycol fragment.

The ratio of the average protons signal intensities of oligoethylene glycol (3.04, 3.60–3.61 ppm) and methyl and methylene substituents of the polymer methacryloyl fragment (3.34 and 4.04 ppm) was used for the calculation of the copolymer compositions. The molar content of the betulin methacrylate units determined using ^1^H NMR spectra was equal to 10.4 mol% and 15.8 mol% for Bet-1 and Bet-2, respectively. These results were close to the HPLC data: 9.3 mol% and 13.3 mol% for Bet-1 and Bet-2, respectively (Figure S1 and Table S1).

**Figure 3:**
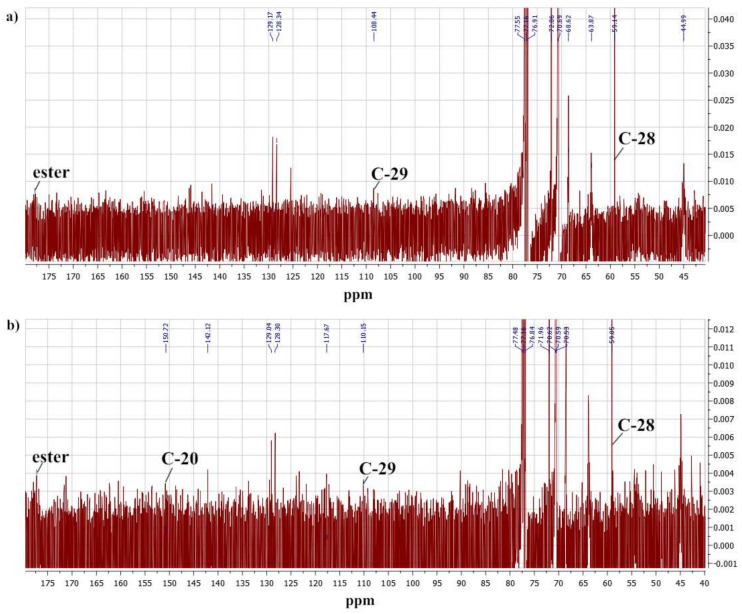
Fragments of the ^13^C NMR spectra of Bet-1 (**a**) and Bet-2 (**b**) copolymers.

The ^13^C NMR spectra of the copolymers also corresponded to their proposed structures (Figure 3). Low-intensity signals in the range from 10 to 50 ppm and signals at 128.3 and 129.1 ppm were due to the betulin skeleton and olefinic carbon atoms, respectively. The singlet at 117.98 ppm corresponded to the nitrile group of the terminal residue of the polymerization initiator. Signals in the 176.6–177.8 ppm region of the spectra reflected the carbon atoms of the carbonyl groups. The most intense signals are typical for oligoethylene glycol and carbons of the main chain of macromolecules.

The molecular weight of the polymers was determined using an UV spectral assay of the residual fragment (trithiocarbonate group) of the initiator in the macromolecule [36]. Table 1 presents the characteristics of the synthesized copolymers.

Table 1 shows the CMC data calculated for the Bet-1 and Bet-2 copolymers using the results of the fluorescent analysis with pyrene (Figure S2).

### 2.2. Properties of Cerium Oxide Nanoparticles

#### 2.2.1. Physicochemical Properties

**Figure 4:**
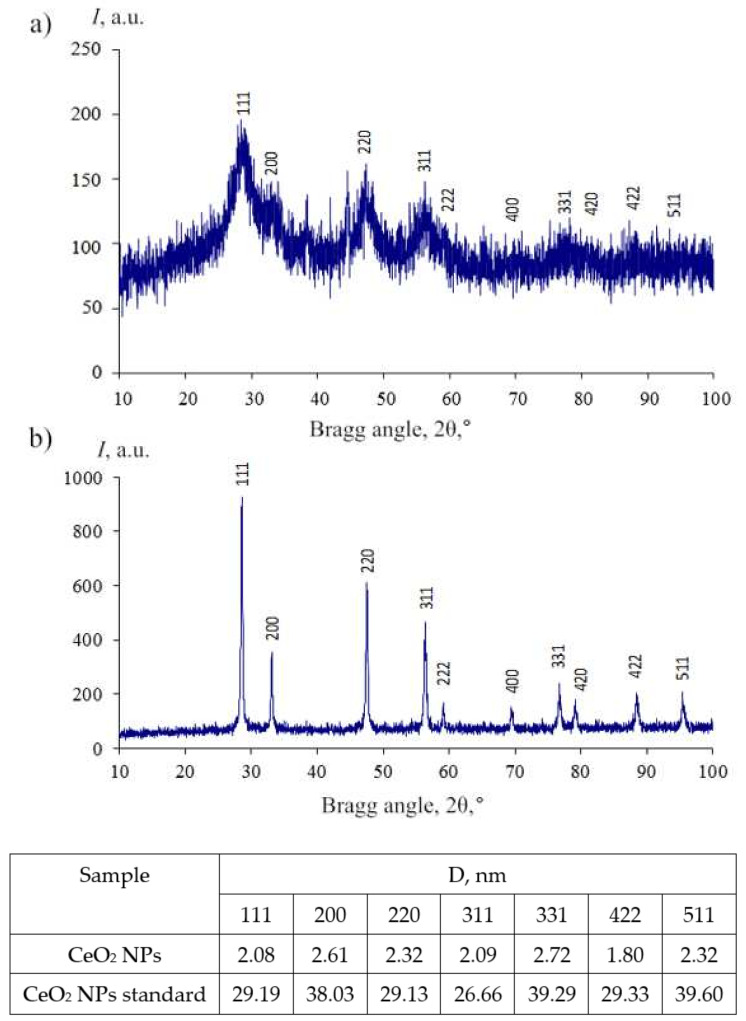
Powder XRD patterns of the cerium oxide nanoparticles modified by maltodextrin (**a**) and a standard sample of cerium oxide nanoparticles (**b**).

The PXRD pattern of the obtained nanoceria had broad reflections characterized by the nanosized cubic fluorite type structure of CeO_2_ (JCPDS no. 34–0394, space group Fm3m), and all reflections were the same as those of the standard CeO_2_ NPs (Figure 4).

The average powder size (D) was estimated using the Scherrer Equation (1) from the powder XRD pattern:

(1) $$D=\frac{k\lambda }{\beta \cos\theta }$$

where λ is the wavelength of the X-ray and equals 1.5056 Å, k is 0.89, β is the half-peak width of the diffraction peak, and θ is the Bragg angle.

The small calculated values of the average powder size (2–3 nm) determined using powder XRD analysis were in good agreement with the BET data of the powders. The specific surface area of the powder was 284.975 m^2^/g, the pore volume was 0.149 cm^3^/g, and the pore diameter was 1.120 nm; this characterized the high dispersity of the powder.

The immobilization of maltodextrin on the surface of CeO_2_ NPs was demonstrated using the FTIR spectrum, where stretching and skeletal vibration bands for C-O and O-H bonds were observed in the regions of 1080–1020 cm^−1^ and 3400 cm^−1^, respectively (Figure S3).

The zeta potential of the CeO_2_ NPs modified using maltodextrin was equal to −16.55 ± 2.18 mV in 20 mg/% PBS saline solution at pH 7.41.

**Figure 5:**
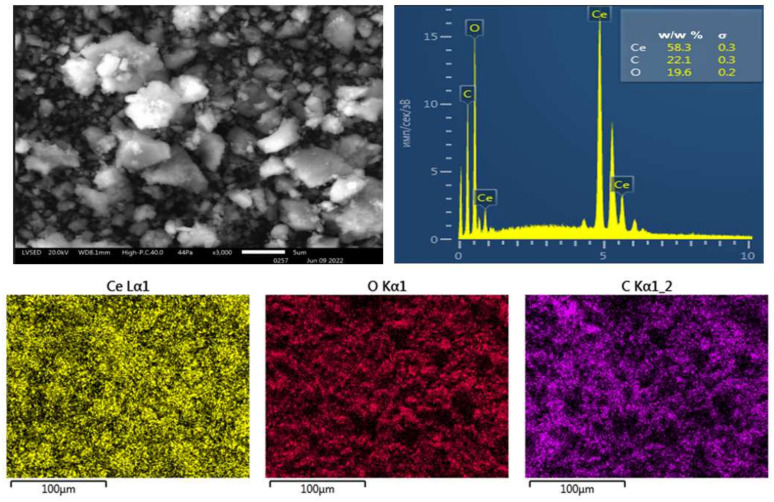
SEM images and EDX spectra of the CeO_2_ NPs modified using maltodextrin.

Figure 5 shows a typical SEM image of the obtained nanoceria, which was placed on an aluminum substrate. Strong ionization during the SEM imaging on aluminum foil led to the aggregation of nanoparticles during the analysis due to maltodextrin being immobilized on the CeO_2_ NPs surface.

**Figure 6:**
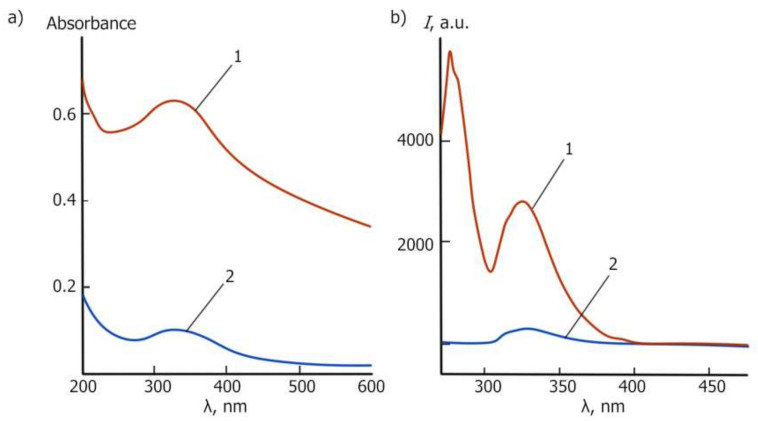
Spectra of the CeO_2_ NPs stabilized using maltodextrin with an average size equal to 2–5 nm (PXRD) (curve 1) and the standard CeO_2_ NPs sample with an average size equal to 40 nm (PXRD) (curve 2): (**a**) UV-vis spectra of 20 mg/% dispersions in methanol; (**b**) fluorescent spectra of 5 mg/% dispersion, λ_ex_ = 265 nm.

The optical properties of the colloidal solution of CeO_2_ NPs were determined using UV-vis spectroscopy. A prominent absorbance peak at about 327 nm was observed, which could be ascribed to the charge transfer transitions from O 2p to Ce 4f, as can be seen in Figure 6a. Figure 6b presents the photoluminescence spectra upon excitation at 265 nm.

The emissions at 275 nm and 324 nm were probably due to 5d → 4f radiative transitions in Ce^3+^ ions. The significant increase in the emission intensity at 324 nm may indicate an increase in the fraction of Ce^3+^ ions on the surface of the nanoparticles. This result may also indicate a smaller nanoparticle size and its promising use as mimetic redox enzymes.

**Figure 7:**
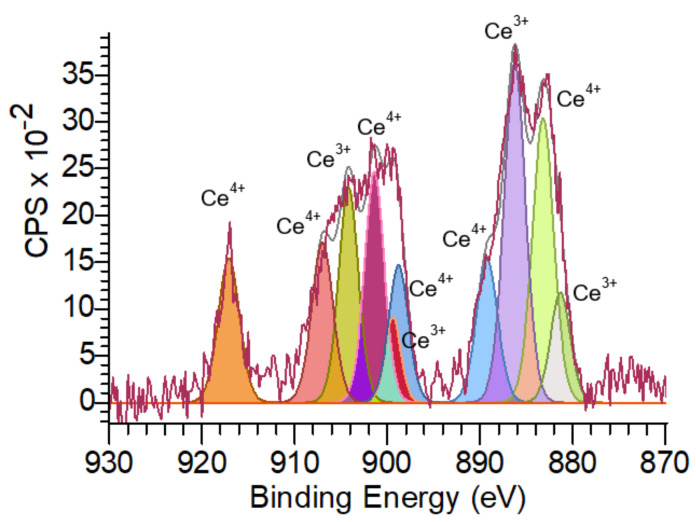
X-ray photoelectron Ce 3*d* spectrum of CeO_2_ NPs.

The Ce 3*d* XPS spectra shown in Figure 7 demonstrated the presence of a mixed valence state (Ce^3+^ and Ce^4+^).

The spectrum of Ce 3*d* revealed that most of the cerium cations were Ce^4+^ (the proportion was 60%), although Ce^3+^ species were also present (the proportion was 40%).

#### 2.2.2. The Activity of Ceria Nanoparticles Assayed Using Cytochrome C

Oxidation properties of ceria nanoparticles that can change the ratio of Ce^3+^/Ce^4+^ were tested using the interaction of cytochrome *c* with a redox system of porphyrin heme (cyt Fe^3+^/cyt Fe^2+^).

The visible absorption spectrum of cyt *c* is sensitive to the spin and coordination states of the heme iron [37], and therefore, was used to examine the activity of cyt *c* after interactions with ceria nanoparticles [37,38,39]. In aqueous solutions, ferricytochrome *c* (Fe^3+^) exhibits two major bands, namely, the intense Soret band at 410 nm and the mild diffuse β band at 530 nm (Q-band), while ferrocytochrome *c* (Fe^2+^) exhibits three bands, namely, the Soret band at 416 nm, the α band at 550 nm, and the β band at 520 nm (Q-bands) [40].

Ferrocytochrome *c* (Fe^2+^) was obtained via the interaction of ferricytochrome *c* (Fe^3+^) with ascorbic acid, which was oxidated to dehydroascorbic acid (Figure S4, Table S2). The absorption of the band at 270 nm that is typical for ascorbic acid was decreased, but the bands belonging to cyt *c* in the region 520–560 nm (two sharp bands at 520 and 550 nm) appeared at the same time and the Soret band shifted to 415 nm.

**Figure 8:**
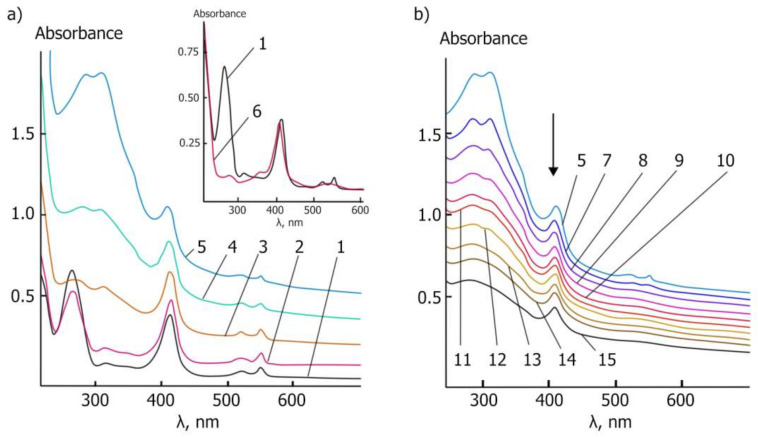
UV-vis spectrum analysis of cytochrome *c* interaction with CeO_2_ NPs. (**a**) The changes in the UV-vis spectra of the CeO_2_ NPs and 0.0334 mM ferrocytochrome *c* (Fe^2+^) reaction mixture in PBS saline (pH 7.4) with respect to the concentration of CeO_2_ NPs. The curves are signified as follows: 1—0 mg/mL, 2—0.25 mg/mL, 3—0.5 mg/mL, 4—0.75 mg/mL and 5—1 mg/mL. Spectra of the initial ferrocytochrome *c* (curve 1) and ferricytochrome *c* (curve 6) are presented in the inset. (**b**) Changes in the UV-vis spectra of CeO_2_ NPs (1 mg/mL) and 0.0334 mM ferrocytochrome c (Fe^2+^) reaction mixture over time (Table 2).

The dependence of the shift and absorbance of Soret and Q-bands on CeO_2_ NPs concentration is presented in Figure 8a and Table 2. The absorption of all bands increased, the blue shift of the Soret band was observed, and a weak diffuse band at 518 nm appeared. The strong increase in all the bands’ absorptions may be explained not only by the oxidation of ferrocytochrome *c* (Fe^2+^) but also by the formation of double-decker cerium porphyrin complexes in the porphyrin heme of cyt *c*, such as double-decker cerium tetra-(15-crown-5)-phtalocyaninate [41].

The UV-vis spectra monitoring the reaction mixture of ferrocytochrome *c* (Fe^2+^) and CeO_2_ NPs over time showed that the Soret band absorption decreased and shifted from 413–414 nm to 408 nm. The Q-bands changed dramatically, and one diffuse band appeared at 520 nm with a decrease in absorbance from 0.633 to 0.226 (Figure 8b, Table 2).

These results demonstrated the ability of ceria nanoparticles to take part in redox reactions with metalloproteins. Therefore, the higher concentration of cerium oxide nanoparticles (the higher surface area) caused the stronger oxidation of ferrocytochrome c (Fe^2+^) to ferricytochrome c (Fe^3+^).

The study of the dynamics of cyt *c* (Fe^2+^) oxidation at the concentration of cerium oxide nanoparticles equal to 1 mg/mL over time showed a decrease in the diffuse Q-band intensity from 0.633 to 0.226 within 45 min, which could characterize the destruction of double-decker cerium porphyrin complexes in the oxidative process and adsorption of cyt *c* (Fe^3+^) on the CeO_2_ NPs.

These results demonstrated the ability of cerium nanoparticles to participate in metalloprotein oxidation reactions, causing a pro-oxidant effect.

### 2.3. The Assessment of Biological Activity In Vitro

The classic molecules that are involved in antioxidation, energy metabolism, and reductive biosynthesis are nicotinamide adenine dinucleotide (NAD^+^), reduced nicotinamide adenine dinucleotide (NADH), nicotinamide adenine dinucleotide phosphate (NADP^+^), and reduced nicotinamide adenine dinucleotide phosphate (NADPH) [42]. It is important to estimate the ratios of NAD^+^/NADH and NADP^+^/NADPH since they can affect antioxidant enzymatic activities and play major roles in oxidative stress.

In this study, we investigated the activity of lactate dehydrogenase (LDH) and aldehyde dehydrogenase (AlDH), which was determined using the ratio NAD^+^/NADH, as well as the activity of glutathione reductase (GR) and glucose-6-phosphate dehydrogenase (G6PDH), which was determined using the ratio NADP^+^/NADPH. Moreover, we investigated the activity of SOD and catalase since they are also important antioxidant components. All biochemical indexes were analyzed in comparison with the control.

Table 3 presents data on the assessment of the specific activity of GR and G6PDH enzymes, as well as SOD and catalase.

The antioxidant properties of the studied copolymers were reflected in a dose-dependent increase in the specific activity of SOD and glucose-6-dehydrogenase in phosphate buffer saline (PBS) at pH 7.4 (Table 3). The SOD activity increased by 10–30% in a dose-dependent manner. The G6PDH activity increased more strongly from 21 to 51% (Bet-1) and 42 to 120% (Bet-2), while the specific activity of catalase practically did not change for Bet-1 and changed slightly for Bet-2 (±10%). The GR activity changed significantly from 9 to 132% only for the copolymer Bet-2 with a higher fraction of the betulin-containing fragment, depending on the dose.

The effect of CeO_2_ NPs on the SOD-specific activity was dose-independent and the increase was 27–33%, while catalase and GR increased in a dose-dependent manner from 4 to 35% and 0 to 18%, respectively (Table 3). An abnormally high increase in G6PDH activity from 44 to 176% under the action of CeO_2_ NPs was observed.

The effect on all four antioxidant defense enzymes was more significant when the combination of the Bet-2 copolymer and CeO_2_ NPs in the micellar system was used. The specific activity of SOD increased by more than 30% at all doses, the catalase activity increased dose-dependently from 13 to 45%, and the GR activity increased to 49% at the highest dose. The maximum increase in activity was observed for the G6PDH enzyme, ranging from 54% at a low dose of 20 µL to 151% at a dose of 50 µL.

The specific activity of lactate dehydrogenase in the direct reaction (LDH_dir_) changed insignificantly (by 9–15%) under the action of all studied samples, while the LDH_rev_ activity was above the control group by 10–35% (Table 4). 

The rise of LDH_rev_ activity characterized the increase in the lactic acid content, which was formed mainly by M-subunits of LDH (LDH_rev_) and indicated the predominance of anaerobic processes in the erythrocytes. The specific LDH_rev_-to-LDH_dir_ activity ratio increased from 4.2 (control) to 5.2–5.8 (combination of Bet-2 and CeO_2_ NPs), which reflected the redox balance [NADH]/[NAD^+^] in the direct and reverse reactions under the action of the Bet-2 and CeO_2_ NPs combination. 

It should be noted that the other NAD^+^/NADH-dependent antioxidant defense enzyme AlDH was not significantly changed compared with the control in all cases (Table 4).

Thus, the studied compounds, namely, Bet-2, CeO_2_ NPs, and their mixture, generally influenced the LDH_reverse_ activity. The effect was most strongly manifested in the case of the combination of Bet-2 and CeO_2_ NPs. 

The antioxidant properties of the studied Bet-1 and Bet-2 copolymers, as well as their combination with CeO_2_ NPs, were also evaluated using the biochemical parameters of lipid peroxidation (LPO), such as the level of malonic dialdehyde (MDA) in the erythrocytes, diene and triene conjugates, and Schiff bases (Table 5).

The level of MDA dose-dependently increased by 3–15% compared with the control for copolymers Bet-1 and Bet-2, while it decreased dose-dependently by 3–12% in samples containing cerium oxide nanoparticles. The Bet-1 copolymer caused more significant oxidative stress based on these indexes.

In contrast, CeO_2_ NPs, both in the PBS and in the micellar system with Bet-2, did not have a significant effect on the levels of DC, TC, and SB, which were expressed as either a slight decrease in all of these indexes or as an insignificant change from the control regardless of dose (Table 5).

## 3. Discussion

The results presented in Table 1, Table S1, and Figure S1 show the convenience of the composition-controlled synthesis of betulin-containing copolymers via the radical copolymerization of hydrophobic betulin methacrylate with hydrophilic methoxyoligo(ethylene glycol)methacrylate. This method of synthesis made it possible to obtain well-defined copolymers with desired amphiphilic properties due to the similar reactivity of both types of methacrylic esters in solution homogeneous radical copolymerization [43].

The first feature of the Bet-1 and Bet-2 copolymers properties in aqueous solutions was a significant increase in their solubility compared with pure betulin or betulin methacrylate, which was due to the significant content of hydrophilic oligoethylene glycol chains. Thus, the solubility of betulin was equal to 1 ppm or less, depending on the solvate’s polymorphic form, while the solubility of betulin units in copolymers was 70–100 ppm. The second feature was the ability of Bet-1 and Bet-2 to form micelles in an aqueous medium such as copolymers of hydrophilic methoxyoligo(ethylene glycol) methacrylates and hydrophobic alkyl methacrylates, which have a hydrophobic core and a hydrophilic shell at certain ratios of units [44]. In recent years, such polymer micelles were widely used to obtain metal-containing catalytic systems, including “nanoenzymes” as mimetic redox enzymes in various organic reactions. Metal-containing nanoparticles (for example, palladium (II) or copper (II) [45,46,47]) were embedded in the hydrophobic cores of amphiphilic polymer micelles. Table 1 and Figure S2 data show that both polymers synthesized by us formed micelles in aqueous solutions, and they formed more easily (at a lower concentration) in the case of the Bet-2 copolymer (15 mol% betulin units) compared with the Bet-1 copolymer (10 mol% units of betulin). Micellization of the synthesized copolymers was evaluated using an organic hydrophobic label, namely, pyrene, which confirmed the ability of micelles to involve “external” hydrophobic compounds. The addition of cerium oxide nanoparticle powder to a homogeneous aqueous solution of Bet-1 and Bet-2 copolymers resulted in the formation of a transparent light yellow dispersion of cerium oxide nanoparticles, which was stable for a long time.

Based on these results and the above literature data on the inclusion of metal nanoparticles in polymer micelles, it can be assumed that hydrophobic cerium oxide nanoparticles in aqueous solutions were incorporated into the hydrophobic cores of micelles of betulin-containing polymers.

The data from the biological study showed the antioxidant activity of the synthesized polymers and their conjugates with cerium oxide nanoparticles.

**Figure 9:**
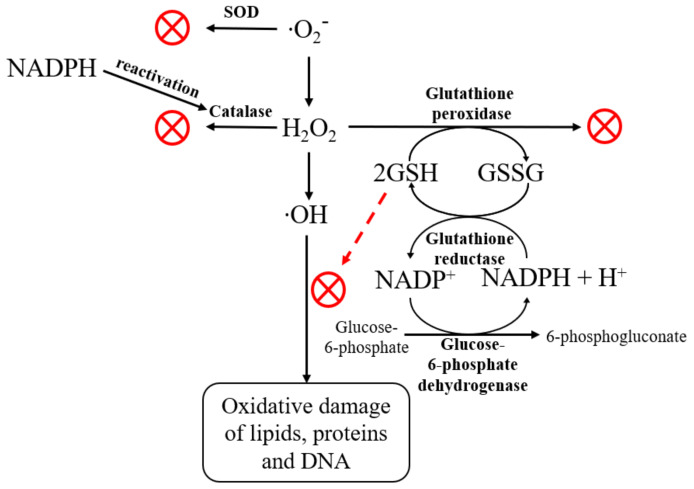
Effects of NADPH and glutathione on the cellular antioxidation capacity due to H_2_O_2_ and ∙OH elimination.

The pentose phosphate pathway (PPP) is the main source of NADPH (an essential cellular reductant) that is required by many essential cellular pathways. Enzymes that utilize the reducing power of NADPH include glutathione reductase, nitric oxide (NO) synthase, cytochrome p450 enzymes, and enzymes in the lipid biosynthesis pathway [42,48]. Figure 9 shows the general scheme of effects of NADPH and glutathione on the cellular antioxidation capacity due to H_2_O_2_ and ∙OH elimination.

If NADPH is limited or decreased, many of these enzyme systems were impaired. The increased NADPH levels under the action of Bet-2 at all doses, CeO_2_ NPs, and the combination of CeO_2_ NPs and Bet-2 at 50 and 100 μL due to GR activity led to an increase in the antioxidant function of the glutathione system. NADPH is the essential reductant required by glutathione reductase to maintain reduced glutathione [44]. Catalase does not require NADPH for its antioxidant function but it has a critical allosteric binding site for NADPH that keeps catalase in its active conformation [49]. SOD does not utilize NADPH. The increase in SOD activity (by 15–37%) under the action of the studied samples was associated with the improved conversion of superoxide to hydrogen peroxide [50].

Then, hydrogen peroxide needs to be metabolized either by catalase or the glutathione system to maintain the redox balance. Hence, the entire antioxidant pathway depends on NADPH. G6PD is the essential regulator of the NADPH that is required by most cellular processes other than lipid production. A key role of glucose-6-phosphate dehydrogenase in NADPH synthesis and antioxidation was further indicated by the finding that the red cells from G6PDH-deficient patients had increased sensitivity [51]. Therefore, the significant increase in G6PDH activity by 50–150% in comparison with the control under the action of all samples demonstrated strong antioxidation capacity in organisms.

**Figure 10:**
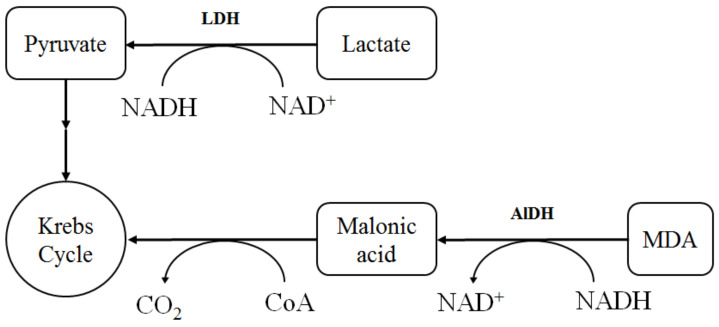
Participation of the NAD^+^/NADH pair in the reactions of some enzymes.

The general scheme of interaction between oxidative and energy metabolism, mediated by the work of NADH/NAD^+^ pyridine nucleotides, is shown in Figure 10.

The increase in the specific LDH_reverse_-to-LDH_direct_ activity ratio after the action of the combination of Bet-2 and CeO_2_ NPs associated with the redox balance of [NADH]/[NAD^+^] in direct and reverse reactions is especially significant for tumor cells. As tumor cells are unable to oxidize NADH using a mitochondrial method, they reoxidize NADH using pyruvate via lactate dehydrogenase; in this case, lactate accumulates in aerobic conditions, although the tricarboxylic acid cycle and the electron transport chain have a normal rate of biochemical reactions that is typical for aerobic conditions in tumor cells. Accordingly, the shift toward the reverse reaction is favorable for tumor cells. It was established that the energy metabolism of tumor cells is determined by the balance between glycolysis and oxidative phosphorylation [52,53]. The metabolic plasticity of CeO_2_ NPs at the dose of 50 μL consisted of the activation of energy metabolism, both in the glycolysis and oxidative phosphorylation (the increase in the LDH_dirt_ and LDH_rev_ activity), which may be useful for the treatment of tumor cells.

## 4. Materials and Methods

### 4.1. Materials

Methacrylic acid (99%), p-toluenesulfonic acid (ACS reagent, ≥98.5%), hydroquinone (ReagentPlus, ≥99%), 4-dimethylaminopyridine (ReagentPlus, ≥99%), dicyclohexylcarbodiimide (99%), 4-cyano-4-[(dodecylsulfanylthiocarbonyl)sulfanyl]pentanoic acid (97%), sodium hydroxide (reagent grade, ≥98%), sodium chloride (ACS reagent, ≥99.0%), toluene (ACS reagent, ≥99.5%), acetone (ACS reagent, ≥99.5%), tetrahydrofuran (ACS reagent, ≥99.5%), chloroform (ACS reagent, ≥99.5%), acetonitrile (for spectroscopy, ≥99.5%), ethylene glycol (anhydrous, 99.8%), pyrene (98%), and hexane (Laboratory Reagent, ≥95%) were purchased from Sigma-Aldrich (Moscow, Russia). 

Methoxyoligoethylene glycol MPEG-350 (Zavod sintanolov LLC, Dzerzhinsk, Russia), maltodextrin (DE = 10–12) (Longcom Enterprise Limited, Hefei, Anhui, China), cytochrome *c* (˃95%, Sigma Aldrich, Moscow, Russia), ascorbic acid (˃99%, Merck, Moscow, Russia), cerium nitrate hexahydrate (Khimkraft LLC, Kaliningrad, Russia), aqueous ammonia 25% (SIGMATEK LLC, Khimki, Russia), ethanol 95% (Acros Organics, Geel, Belgium), and purified water (resistivity ≥18 MΩ·cm, Millipore, Merck, Darmstadt, Germany) were used in the study. 

Betulin was purchased from “NPO Ecodika” (Kirov, Russia) and further purified by boiling with sodium hydroxide (0.5%) in toluene and recrystallized from ethanol or isopropanol. FTIR, ν, cm^−1^: 3470 st (OH), 1640 st (C=C); ^1^H NMR δ, ppm: 4.67 m (1H, =CH_2_), 4.57 m (1H, =CH_2_), 3.78 br. s (1H, 28-CH_2_OH), 3.31 m (1H, 28-CH_2_OH), 3.17 m (1H, 3-CHOH), 2.36 m (1H, 19-CH), 1.66 s (3H, CH_3_), 1.23 s (3H, CH_3_), 0.96 s (3H, CH_3_), 0.94 s (3H, CH_3_), 0.80 s (3H, CH_3_), 0.74 s (3H, CH_3_). ^13^C NMR, δ, ppm: 76.71 (C-3), 109.46 (C-29), 150.24 (C-20), 57.87 (C-28).

### 4.2. Synthesis of Copolymer Conjugates of Methoxyoligo(Ethylene Glycol)Methacrylate and Betulin Methacrylate

#### 4.2.1. Synthesis of Methoxyoligo(Ethylene Glycol)Methacrylate

The synthesis of methoxyoligo(ethylene glycol)methacrylate (MOEGM) was carried out by analogy with the previously developed method [54]. MOEGM was synthesized by the esterification of methacrylic acid with methoxyoligoethylene glycol (3:1 mol) at 120 °C in a toluene solution (toluene content 30.0 wt%) in the presence of p-toluene sulfonic acid (2.0 wt%) as a catalyst and hydroquinone (0.3 wt%) as a polymerization inhibitor. The reaction was carried out in a four-necked reactor equipped with a mechanical stirrer, a thermometer, and a Dean–Stark trap. The resulting water was removed from the reaction zone in the form of an azeotrope with toluene. The duration of the synthesis was 4 h. The resulting reaction mass was diluted with a 10-fold excess of chloroform and washed with a 5% sodium hydroxide solution (taken with a 3% excess relative to the acid groups) to remove unreacted methacrylic acid and hydroquinone. After neutralization, the organic layer was washed several times with saturated sodium chloride solution. The solvent was removed using a rotary evaporator under reduced pressure. The average ethylene glycol units and MOEGM content (98%) of the synthesized monomer product was determined using an assay of C=C double bonds (using bromide-bromate titration) and NMR spectroscopy data. ^1^H NMR (400 MHz, chloroform-d, 25 °C, δ = 7.27 (chloroform)): δ 6.11 (1H, C**H**_2_=), 5.55 (1H, C**H**_2_=), 4.28 (2H, COOC**H**_2_-), 3.71–3.42 (16H, -C**H**_2_O(C**H**_2_C**H**_2_O)_n_CH_3_), 1.93 (3H, CH_2_=C(C**H**_3_)COO^-^).

#### 4.2.2. Synthesis of Betulin Methacrylate

Betulin methacrylate was obtained via the esterification of methacrylic acid. Totals of 4 mmol of betulin, 6 mmol of dicyclohexylcarbodiimide, and 0.2 mmol of 4-dimethylaminopyridine were dissolved in 8 mL of tetrahydrofuran. The mixture was cooled to −20 °C, and a solution of methacrylic acid (9 mmol in 5.3 mL of tetrahydrofuran) was added dropwise to the mixture with vigorous stirring for 2 h. Then, the reaction mixture was kept for 24 h at room temperature. An excess of methacrylic acid was added to the mixture to remove the residue of unreacted dicyclohexylcarbodiimide. The precipitate of dicyclohexylurea was removed via centrifugation. Then, 13 mL of acetone was added to the reaction mixture, after which the product was precipitated in 260 mL of distilled water, separated via centrifugation, washed with distilled water on a filter, and dried under vacuum to constant weight at a temperature of 75 °C. The resulting product was recrystallized from hexane to remove the betulin dimethacrylate impurities. ^1^H NMR (400 MHz, chloroform-d, 25 °C, δ = 7.27 (chloroform)): 6.09–6.12 (1H, =CH_2_, methacr.), 5.53–5.56 (1.01 H, =CH_2_, methacr.), 4.68–4.70 (1H, C-30), 4.59–4.60 (0.99H, C-30), 4.33 (0.99 H, C-28), 3.91–3.94 (1H, C-28), 3.17–3.21 (1.01 H, C-3), 2.43–2.50 (0.99 H, C-19).

#### 4.2.3. Synthesis of Copolymers

The copolymer was obtained using RAFT polymerization. 4-Cyano-4-(dodecylsulfanylthiocarbonyl)sulfanyl pentanoic acid was used as the reversible chain transfer agent. The synthesis was carried out in toluene at a temperature of 80 °C and azobisisobutyronitrile was used as an initiator. The initial total concentration of monomers was 20 wt%, while the mass ratios of betulin methacrylate and MPEGMA 500 were 10:90 and 15:85 for copolymers Bet-1 and Bet-2, correspondingly. The molar ratio of monomer/RAFT-agent/initiator was 600/4/1 (mol). Polymerization was carried out for 4 h.

During the copolymerization, the concentrations of both comonomers were determined, which made it possible to calculate the compositions of the copolymers. The adequacy of the method was confirmed using the correspondence between the calculated values of the copolymer compositions determined experimentally via the ^1^H NMR method for the polymer samples isolated from the reaction mixtures.

The concentration of monomers in the initial and final reaction mixtures was determined using HPLC (Figure S1, Table S1). After finishing the polymerization, the polymers were isolated via repeated precipitation with hexane from a toluene solution. The molecular weights of the polymers were determined via UV spectroscopy from the absorption intensity of the trithiocarbonate group in the macromolecule [36]. The UV spectra of the polymers were recorded in acetonitrile.

### 4.3. Synthesis of Cerium Oxide Nanoparticles Stabilized Using Maltodextrin

Maltodextrin-coated cerium oxide nanoparticles were synthesized according to the methods [55] with minor modifications. Ammonium hydroxide solution (25%) was slowly added dropwise to a mixture containing 10 mL of a 1 M solution of cerium nitrate hexahydrate (4.35 g in 10 mL) and 20 mL of a 0.1 M solution of maltodextrin at room temperature in up to pH 10.0 with vigorous stirring. The resulting dispersion was stirred for 24 h. The color changed from clear and colorless to light yellow, then to dark brown. The precipitate was centrifuged and successively washed several times with hot water and ethanol; then, the yellow-brown precipitate was dried under vacuum.

### 4.4. Analysis Methods

#### 4.4.1. Determination of the Critical Micelle Concentration (CMC)

Micellization was studied in accordance with the procedure used in [56]. The CMC of polymers was determined by fluorimetry using pyrene as a fluorophore probe (Figure S2). Fluorescent spectra were obtained using an RF-6000 spectrofluorometer (Shimadzu, Kyoto, Japan) with an excitation wavelength of 335 nm, emission in the field of 350–500 nm, and using a 10 mm-thick cuvette at 25 °C. Polymers were dissolved in an aqueous solution of pyrene (2 × 10^−7^ M) until they reached their predetermined concentration. The obtained ratios of the intensities of the first (I_1_ ~ 373 nm) and third (I_3_ ~ 384 nm) emission bands of pyrene were presented as a function of the polymer concentration. The CMC value was determined as the concentration at which the I_1_/I_3_ ratio began to sharply decrease.

#### 4.4.2. SEM and EDXMA Studies

Microphotographs of samples were obtained via scanning electron microscopy (SEM) on a JSM-IT300LV (JEOL, Tokyo, Japan) microscope with an electron beam diameter of about 5 nm and a probe current below 0.5 nA (operating voltage 20 kV). The surface topography of the samples was studied using low-energy secondary electrons and backscattered electrons. The elemental composition of the samples was studied using X-ray microprobe analysis (XRM) with an X-MaxN 20 detector (Oxford Instruments, Oxford, UK).

#### 4.4.3. Specific Area Estimation

The specific surface area of the powder materials was determined using the method of static vacuum volumetry (analysis of the surface area, micropore size, and chemisorption using “Autosorb iQ C” (Quantachrome Instruments, Boynton Beach, FL, USA)). Before the measurements, the samples were degassed under a dynamic vacuum (base pressure = 1.33 × 10 Pa) at a temperature of 120 °C for 3 h. The specific surface area of the powder was estimated via the Brunauer–Emmett–Teller (BET) method using data taken in the range 0.05 < *p*/*p_o_* < 0.35.

#### 4.4.4. Surface Charge and Dynamic Light Scattering Measurements

The surface charge and average hydrodynamic diameter of the samples were measured using a NanoBrook Omni (Brookhaven Instruments, NY, USA). The zeta potential was determined via phase analysis electrophoretic light scattering (PALS). The Smoluchowski model was used to convert the electrophoretic mobility values to the zeta potential values.

The hydrodynamic diameter of the nanoparticles was determined using dynamic light scattering (DLS) in the mode of multimodal analysis of the correlation function. The measurements were carried out at 25 ± 0.1 °C at an angle of 90° in the range from 0.1 to 5000 nm in polystyrene cuvettes (1 cm). The accumulation time of the correlation function was 180 sec (n = 10).

#### 4.4.5. NMR

^13^C and ^1^H NMR spectra were recorded at 100 and 400 MHz, respectively, on a Jeol JNM ECX-400 spectrometer (Jeol Ltd., Tokyo, Japan) using CDCl_3_ (50 mg/0.6 mL) as the solvent at 25 °C. As reference signals for the correction of the scale of chemical shifts, we used the signals of residual protons of the solvent (δ = 7.26 ppm) and carbon atoms of CDCl_3_ (δ = 77.16 ppm) for the ^1^H and ^13^C nuclei, respectively, as presented in [57].

#### 4.4.6. Specific Area Estimation

FTIR spectra in the 400–4000 cm^−1^ range were measured using an IR Prestige-21 FTIR spectrometer (Shimadzu, Kyoto, Japan) equipped with a KBr beam splitter. A pellet from a well-dried KBr was prepared according to standard cold pressing. The resolution was 0.5 cm^−1^. The number of scans was 45.

#### 4.4.7. UV Spectrophotometry

UV spectra were recorded using a UV-1800 (Shimadzu USA Manufacturing Inc., Canby, OR, USA).

#### 4.4.8. Powder X-ray Diffractometry

Powder X-ray diffraction patterns were obtained using an XRD-6000 X-ray diffractometer (Shimadzu, Kyoto, Japan) at 295(2) K with Cu Kα radiation (λ = 1.5418 Å) in the Bragg–Brentano reflection geometry. The samples were collected in the 2θ range between 5 and 50° with steps of 0.026° and 100 s of step size using a scan speed of 0.067335°/s. In the X-ray diffraction patterns of amorphous samples, there were diffraction peaks at 37.5° and 44.0°, which referred to the cuvette material.

#### 4.4.9. HPLC

Monomer conversion was determined via HPLC using a chromatographic system “Shimadzu Prominence” (Shimadzu, Kyoto, Japan) equipped with refractometric and matrix detectors, a thermostat, and a Kromasil column (100-5-C18 4.6 × 250 mm). Acetonitrile was used as the eluent at a flow rate of 0.9 mL/min and a column temperature of 55 °C.

#### 4.4.10. XPS Analysis

XPS measurements were carried out using an ultrahigh vacuum complex Multiprobe RM (Omicron Nanotechnology GmbH, Taunusstein, Germany). Photoemission was excited under non-monochromatized Mg Kα radiation (energy was equal to 1253.6 eV, linewidth was equal to 0.7 eV). The analysis area diameter was 3 mm. The limiting detectable concentration of elements was determined using the signal-to-noise ratio in the photoelectron spectra and was 0.1–1 at%. Ce 3*d* high-resolution analysis was recorded at an analyzer pass energy of 30 eV and an energy step of 0.2 eV. Mathematical processing of the spectra was carried out using the software SDP version 4.3 and CasaXPS.

### 4.5. Biological Experiments

#### 4.5.1. Biological Activity

Male Wistar rats (200–250 g) were involved in the study. The animals were purchased from the Animal Breeding Facilities “Andreevka” Federal State Budgetary Institution of Science “Scientific Center for Biomedical Technologies” of the Federal Medical and Biological Agency (Andreevka, Moscow Oblast, Russia). The animals were handled humanely, kept in plastic suspended cages, and placed in a well-ventilated and hygienic rat house under suitable room temperature (27 ± 2 °C) and humidity conditions. They were given food and water ad libitum and subjected to a natural photoperiod cycle of 12 h light and 12 h dark. The animals were allowed two weeks of acclimatization before the commencement of all animal model experiments in the study.

All blood withdrawals from the animals for the experiment were performed under anesthesia, with all efforts made to minimize suffering.

The animal study was conducted according to the guidelines of the Declaration of Helsinki and approved by the Local Ethics Committee of Privolzhsky Research Medical University, Russian Federation (protocol no. 1 from 18 January 2021). 

*In vitro* biological analysis was performed using blood stabilized with sodium citrate (1:9). Erythrocytes were washed twice with 0.9% NaCl by centrifugation for 10 min at 1600×*g*. The intensity of lipid peroxidation (LPO) was estimated using the MDA level in erythrocytes in accordance with the methods of Uchiyama and Mihara [58]. Determination of the content of diene conjugates (DC), triene conjugates (TC), and Schiff bases (SB) in the blood plasma was carried out according to the Khyshiktuev method [59]. Superoxide dismutase SOD activity (EC 1.15.1.1) was measured in erythrocytes using the inhibition of adrenaline auto-oxidation [60]. Catalase activity (EC 1.11.1.6) was determined via spectrophotometry based on the decomposition of hydrogen peroxide by the catalase [61]. Glutathione reductase GR activity (EC 1.8.1.7) was studied via spectrophotometry based on the oxidized glutathione reduction [62]. The activity of glucose-6-phosphate dehydrogenase G6PDH (EC 1.1.1.49) was determined in the hemolysate of erythrocytes via spectrophotometry based on glucose-6-phosphate oxidation to phosphoglucolactone with the formation of reduced nicotinamide adenine dinucleotide phosphate (NADPH) [63]. The energy metabolism in erythrocytes was studied using the catalytic activity of lactate dehydrogenase LDH (EC 1.1.1.27) in direct (LDH_direct_, substrate—50 mM of sodium lactate) and in reverse (LDH_reverse_, substrate—23 mM of sodium pyruvate) reactions [64]. The activity of aldehyde dehydrogenase (EC 1.2.1.3) was estimated spectrophotometrically in accordance with previous methods [65]. The specific activity of the enzymes was calculated from the protein concentration analyzed via the modified Lowry method [66].

#### 4.5.2. Samples for Biological Experiments

Samples were prepared for analysis of the biological activity (Table 6).

A total of 20 µL, 50 µL, and 100 µL of the resulting solutions were mixed with 1 mL of intact rat blood and incubated for 10 min at room temperature. The mixture was then centrifuged at 1500 rpm for 10 min to obtain the plasma and red blood cells.

#### 4.5.3. Statistical Analysis

Statistical data processing was performed using Statistica 6.0 software (StatSoft Inc., Tulsa, OK, USA). The normality of the distribution of the results was shown using the Shapiro–Wilk test. The significance of differences between groups was assessed using Student’s t-test and one-way analysis of variance (ANOVA). The differences were considered statistically significant at *p* < 0.05.

If the distribution of at least one of the populations was not normal, nonparametric analysis methods were used for the comparison. The non-parametric Kruskal–Wallis test was used for multiple comparisons of independent groups. The Mann–Whitney test was used for multiple comparisons for 2 groups.

## 5. Conclusions

In this work, two new copolymers with a hydrophobic unit, namely, betulin methacrylate, were synthesized for the treatment of diseases caused by oxidative stress. The hydrophilic methoxyoligo(ethylene glycol)methacrylate unit of the copolymer allowed for improving the solubility in water (70–100 ppm in terms of betulin) compared with betulin (1 ppm). The micellar system of MOEGM-BM copolymers contained cerium oxide nanoparticles, which exhibited the properties of a mimetic of antioxidant enzymes.

The work demonstrated a dose-dependent increase in the activation of antioxidant defense enzymes, namely, SOD, catalase, GR, and G6PDG, under the action of compositions, including new copolymers MOEGM-BM and CeO_2_ NPs. This may be explained by the role of NADPH in the antioxidant system.

Betulin-containing copolymers conjugates with CeO_2_ NPs can be used for the design of new components of medicines for the treatment of diseases that are caused by oxidative stress and for the preparation of biomimetic medical products with controlled antioxidant properties.

## Figures and Tables

**Table 1:** Characteristics of Bet-1 and Bet-2 copolymers.

Sample	Copolymer Composition		Average Units		Molar Weight (UV Spectral Assay)	CMC, wt%
	Betulin Methacrylate Units, mol%	MOEGM Units, mol%	x	y		
Bet-1 ^1^	10.4	89.7	219.1	25.3	115 000	0.00010
Bet-1 ^2^	9.3	90.8	222.1	22.6		
Bet-2 ^1^	15.8	84.2	132.4	24.8	75 000	0.00007
Bet-2 ^2^	13.3	86.7	136.7	20.9		

^1^ Via HPLC data. ^2^ Via ^1^H NMR data.

**Table 2:** Data from the UV-vis spectra of CeO_2_ NPs (0.25, 0.5, 0.75, 1 mg/mL) and 0.0334 mM ferrocytochrome c (Fe^2+^) reaction mixture in PBS saline (pH 7.4).

No. of Curves	CeO_2_ NPs Concentration, mg/mL	Time, min	λ, nm (A)		
			Cyt *c*		
			γ-Band	β-Band	α-Band
1	0	0	415.0 (0.384)	520.5 (0.043)	549.5 (0.066)
2	0.25	0	414.0 (0.479)	520.5 (0.124)	549.5 (0.155)
3	0.50	0	413.5 (0.651)	520.0 (0.279)	549.5 (0.298)
4	0.75	0	412.0 (0.835)	520.0 (0.457)	549.5 (0.459)
5	1.00	0	409.00 (1.055)	520.0 (0.633)	549.5 (0.626)
7		5	407.0 (0.964)	520.0 diff. ^1^ (0.583)	-
8		10	408.0 (0.891)	520.0 diff. (0.539)	-
9		15	407.5 (0.806)	520.0 diff. (0.486)	-
10		20	407.5 (0.734)	520.0 diff. (0.439)	-
11		25	407.5 (0.694)	520.0 diff. (0.397)	-
12		30	408.0 (0.638)	520.0 diff. (0.363)	-
13		35	408.0 (0.573)	520.0 diff. (0.322)	-
14		40	408.0 (0.520)	518.5 diff. (0.285)	-
15		45	408.0 (0.427)	518.5 diff. (0.226)	-

^1^ diff.—diffuse peak.

**Table 3:** The activity of antioxidant defense enzymes in the blood of rats under the action of the studied compounds at the doses of 20, 50, and 100 µL of the sample per 1 mL of blood as a percentage relative to the control (n = 3).

Sample in PBS, pH 7.4 (mg/mL)	Dose, µL/mL	Index							
		GR		G6PDH		SOD		Catalase	
		NADPH/min·mg Protein	% of Control	NADPH/min·mg Protein	% of Control	a.u./min·mg Protein	% of Control	μmol H_2_O_2_/ min·mg Protein	% of Control
Control (100%)	-	90.21 ± 1.49	100.0	40.86 ± 1.17	100.0	959.23 ± 11.28	100.0	35.76 ± 1.26	100.0
Bet-1	20	89.03 ± 4.05	98.7	49.39 ± 1.68	120.9	1052.01 ± 9.61	109.7	35.33 ± 0.92	98.8
	50	89.43 ± 2.42	99.1	58.73 ± 1.55	143.7	1111.16 ± 12.97	115.8	35.49 ± 1.25	99.3
	100	93.73 ± 4.54	103.9	61.60 ± 2.35	150.8	1246.47 ± 8.904	130.0	38.90 ± 2.12	108.8
Bet-2	20	98.12 ± 0.91	108.8	58.13 ± 1.41	142.3	1086.69 ± 10.57	113.3	32.40 ± 1.11	90.6
	50	114.05 ± 3.74	126.4	80.48 ± 1.04	197.0	1161.87 ± 20.93	121.1	33.80 ± 0.40	94.5
	100	208.91 ± 4.64	231.6	90.03 ± 1.81	220.4	1281.13 ± 10.89	133.6	36.43 ± 0.85	101.9
CeO_2_ NPs	20	88.00 ± 0.85	97.6	58.92 ± 0.82	144.2	1215.40 ± 15.83	126.7	37.16 ± 1.50	103.9
	50	100.45 ± 2.20	111.4	99.15 ± 1.16	242.7	1273.05 ± 26.20	132.7	46.60 ± 3.10	130.3
	100	105.99 ± 1.43	117.5	112.58 ± 0.98	275.6	1272.14 ± 34.47	132.6	48.11 ± 3.90	134.6
Combination of Bet-2 and CeO_2_ NPs	20	83.09 ± 0.92	92.1	62.76 ± 1.70	153.6	1268.97 ± 6.66	132.3	40.40 ± 3.25	113.0
	50	102.60 ± 3.22	113.7	102.54 ± 2.82	251.0	1301.67 ± 12.54	135.7	51.72 ± 0.39	144.6
	100	134.19 ± 6.04	148.8	95.87 ± 1.41	234.7	1318.35 ± 25.60	137.4	46.92 ± 4.29	131.2
Kruskal–Wallis test		*p* ≤ 0.0001		*p* ≤ 0.0001		*p* ≤ 0.0001		*p* = 0.002	

**Table 4:** The specific activity of aldehyde dehydrogenase and lactate dehydrogenase in direct and reverse reactions under the action of the studied compounds (% of control), n = 3, *p* < 0.001.

Sample in PBS, pH 7.4 (mg/mL)	Dose, µL/mL	Index (% of Control)						LDH_rev_/ LDH_dir_, ([NADH]/ [NAD^+^])
		AlDH		LDH_dir_		LDH_rev_		
		nmol NADH/ min·mg Protein	% of Control	nmol NAD^+^/ min·mg Protein	% of Control	nmol NADH/ min·mg Protein	% of Control	
Control (100%)	-	40.07 ± 1.76	100.0	42.48 ± 1.15	100.0	176.47 ± 3.66	100.0	4.2
Bet-1	20	36.79 ± 2.45	91.8	36.78 ± 0.66	86.6	198.37 ± 0.87	112.4	5.4
	50	39.81 ± 0.95	99.4	40.00 ± 1.64	94.1	156.23 ± 3.09	88.5	3.9
	100	49.92 ± 1.48	124.6	41.95 ± 1.58	98.7	149.09 ± 3.62	84.5	3.6
Bet-2	20	35.69 ± 1.21	89.1	37.94 ± 0.98	89.3	197.38 ± 2.01	111.8	5.2
	50	40.19 ± 2.01	100.3	39.73 ± 0.99	93.5	212.54 ± 5.22	120.4	5.3
	100	38.60 ± 2.50	96.3	46.23 ± 1.00	108.8	204.18 ± 6.30	115.7	4.4
CeO_2_ NPs	20	37.52 ± 1.52	93.6	39.19 ± 0.63	92.2	221.28 ± 1.19	125.4	5.6
	50	44.01 ± 1.82	109.8	54.30 ± 0.78	127.8	237.24 ± 1.70	134.4	4.4
	100	46.19 ± 1.50	115.3	42.99 ± 0.97	101.2	229.69 ± 3.63	130.2	5.3
Combination of Bet-2 and CeO_2_ NPs	20	40.75 ± 2.30	101.7	35.61 ± 0.68	83.8	205.94 ± 2.95	116.7	5.8
	50	39.36 ± 0.60	98.2	39.61 ± 1.19	93.2	217.31 ± 3.84	123.1	5.5
	100	34.18 ± 0.73	85.3	40.69 ± 0.85	95.8	217.98 ± 3.71	123.5	5.4
Kruskal–Wallis test		*p* = 0.003		*p* ≤ 0.0001		*p* ≤ 0.0001		

**Table 5:** Levels of malonic dialdehyde, diene and triene conjugates, and Schiff bases in the blood of rats under the action of the studied compounds at the doses of 20, 50, and 100 µL of the sample per 1 mL of blood as a percentage relative to the control (n = 3)^1^.

Sample in PBS, pH 7.4 (mg/mL)	Dose, µL/mL	Concentration, % of Control							
		MDA_er_		DC		TC		SB	
		μmol/L	% of Control	a.u.	% of Control	a.u.	% of Control	a.u.	% of Control
Control (100%)	-	7.28 ± 0.25	100.0	0.67 ± 0.02	100.0	0.27 ± 0.01	100.0	0.14 ± 0.01	100.0
Bet-1	20	7.95 ± 0.10	109.2	0.68 ± 0.01	102.5	0.28 ± 0.01	103.0	0.15 ± 0.01	104.3
	50	8.29 ± 0.03	113.8	0.72 ± 0.01	108.5	0.27 ± 0.01	101.1	0.17 ± 0.01	120.7
	100	8.48 ± 0.17	116.5	0.77 ± 0.01	116.0	0.26 ± 0.02	96.6	0.17 ± 0.01	122.9
Bet-2	20	7.55 ± 0.03	103.7	0.63 ± 0.01	94.6	0.26 ± 0.01	98.1	0.15 ± 0.01	104.3
	50	7.76 ± 0.03	106.5	0.65 ± 0.01	96.7	0.27 ± 0.01	100.7	0.15 ± 0.01	110.0
	100	7.66 ± 0.03	105.1	0.67 ± 0.01	100.6	0.28 ± 0.01	103.4	0.15 ± 0.01	110.0
CeO_2_ NPs	20	7.23 ± 0.24	99.2	0.71 ± 0.01	106.6	0.27 ± 0.01	99.6	0.14 ± 0.01	99.6
	50	6.38 ± 0.10	87.6	0.70 ± 0.01	105.1	0.26 ± 0.01	96.3	0.13 ± 0.01	95.7
	100	6.15 ± 0.06	84.5	0.67 ± 0.10	101.0	0.25 ± 0.01	92.5	0.13 ± 0.01	92.1
Combination of Bet-2 and CeO_2_ NPs	20	7.18 ± 0.09	98.6	0.67 ± 0.01	99.9	0.28 ± 0.01	103.0	0.14 ± 0.01	102.1
	50	6.70 ± 0.27	92.0	0.69 ± 0.04	104.0	0.28 ± 0.01	103.7	0.17 ± 0.01	120.0
	100	6.78 ± 0.09	93.1	0.72 ± 0.01	108.1	0.26 ± 0.01	98.5	0.17 ± 0.01	122.1
Kruskal–Wallis test		*p* ≤ 0.0001		*p* ≤ 0.0001		*p* ≤ 0.0001		*p* ≤ 0.0001	

^1^*p* < 0.05 compared with intact blood (*p* = 0.020); *p* < 0.05 compared with the level of the indicator in the ratio of 20 μL of the sample:1 mL of blood (within the group); *p* < 0.05 compared with the level of the indicator in the ratio of 50 μL of the sample:1 mL of blood (within the group).

**Table 6:** Composition of the samples to measure the biological activity.

Sample	Preparation
Bet-1	1.41 mg of Bet-1 was dissolved in 1 mL of PBS
Bet-2	1.41 mg of Bet-2 was dissolved in 1 mL of PBS
CeO_2_-MD ^1^	0.8 mg of CeO_2_-MD was dispersed in 1 mL of PBS
Mixture of Bet-2 and CeO_2_-MD	0.71 mg of Bet-2 and 0.4 mg of CeO_2_-MD were dispersed in 1 mL of PBS

^1^ MD—maltodextrin.

## Data Availability

The data presented in this study are available upon reasonable request from the corresponding author.

## Supplementary Materials

The following supporting information can be downloaded from https://www.mdpi.com/article/10.3390/molecules27185894/s1. Figure S1: HPL chromatograms for RAFT polymerization control. Table S1: Data from the HPL chromatograms for RAFT polymerization control. Figure S2: Fluorescence spectra of pyrene (2 × 10^–7^ M) at different concentrations of Bet-1 (a) and Bet-2 (b) co-polymers in water at 25 °C. Figure S3: FTIR spectrum of maltodextrin modified using cerium oxide nanoparticles. Figure S4: Oxidation of ascorbic acid in an acidic medium using cytochrome *c*. Table S2: Dynamics of cytochrome *c* reduction using ascorbic acid according to electronic absorption spectra.
