# Supplementary material for: Antioxidant Activity of New Copolymer Conjugates of Methoxyoligo(Ethylene Glycol)Methacrylate and Betulin Methacrylate with Cerium Oxide Nanoparticles In Vitro
Source: Molecules. 2022 Sep 11;27(18):5894. doi: 10.3390/molecules27185894 (PMC9506406; doi:10.3390/molecules27185894)
Supplement: Supplementary file 1 [file molecules-27-05894-s001.zip › molecules-1899378-supplementary.pdf]

Figure S1: HPL chromatograms for RAFT polymerization control.

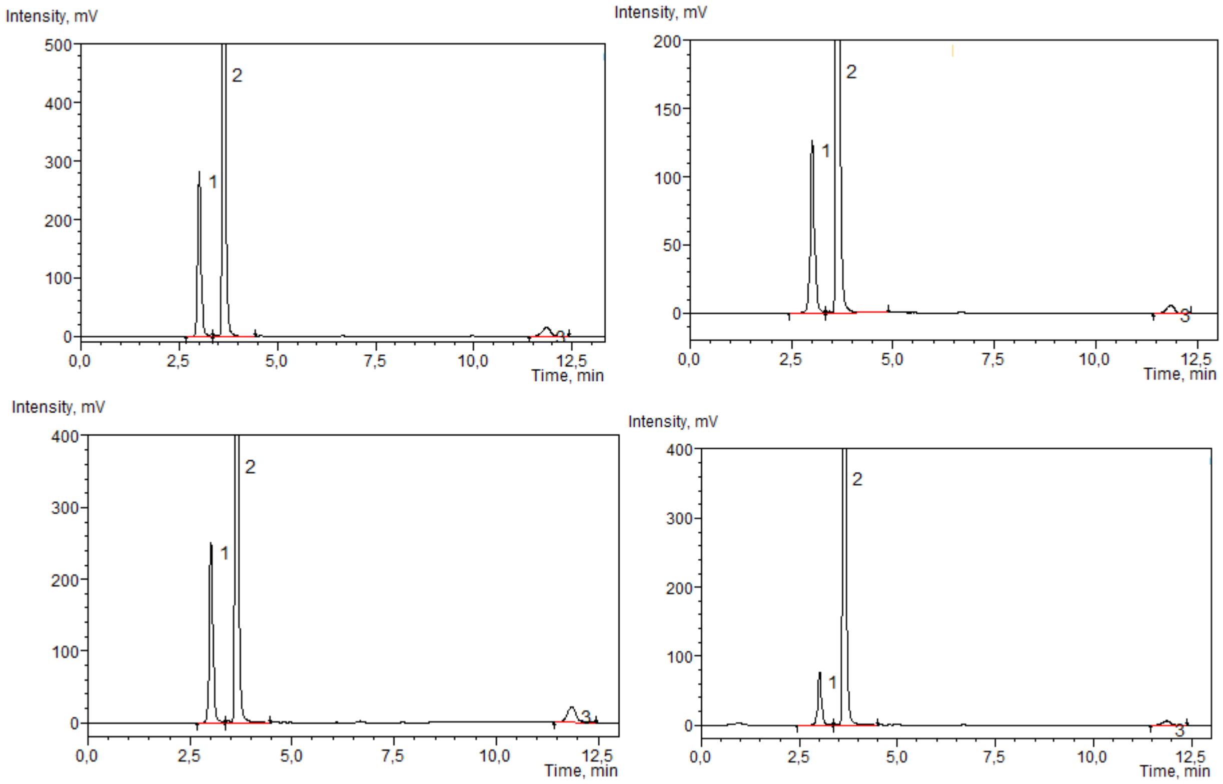

HPL Chromatograms of 1-methoxyoligo(ethylene glycol)methacrylate, 2-toluene, 3-betulin methacrylate. Matrix detector, 235 nm: 1) initial reaction mixture for the synthesis of Bet-1; 2) final reaction mixture for the synthesis of Bet-1; 3) initial reaction mixture for the synthesis of Bet-2; 4) final reaction mixture for the synthesis of Bet-2.

Table S1: Data from the HPL chromatograms for RAFT polymerization control.

Mixture of MPEGMA and BM	Peak	Component	Retention time, min	Area, u.a.	High, mV
Bet-1 before polymerization	1	MPEGMA	3,007	1733741	283395
	2	2-toluene	3,622	6077531	1221162
	3	BM	11,843	215110	14724
Bet-1 after polymerization	1	MPEGMA	3,004	811259	128485
	2	2-toluene	3,619	5971402	1218015
	3	BM	11,844	92941	6363
Bet-2 before polymerization	1	MPEGMA	3,011	1551697	250674
	2	2-toluene	3,628	5970543	1215832
	3	BM	11,853	323222	21955
Bet-2 after polymerization	1	MPEGMA	3,012	500159	76665
	2	2-toluene	3,629	6108897	1239888
	3	BM	11,866	86689	5849

Figure S2: Fluorescence spectra of pyrene (2×10^{-7} M) at different concentrations of Bet-1 (a) and Bet-2 (b) co-polymers in water at 25 °C.

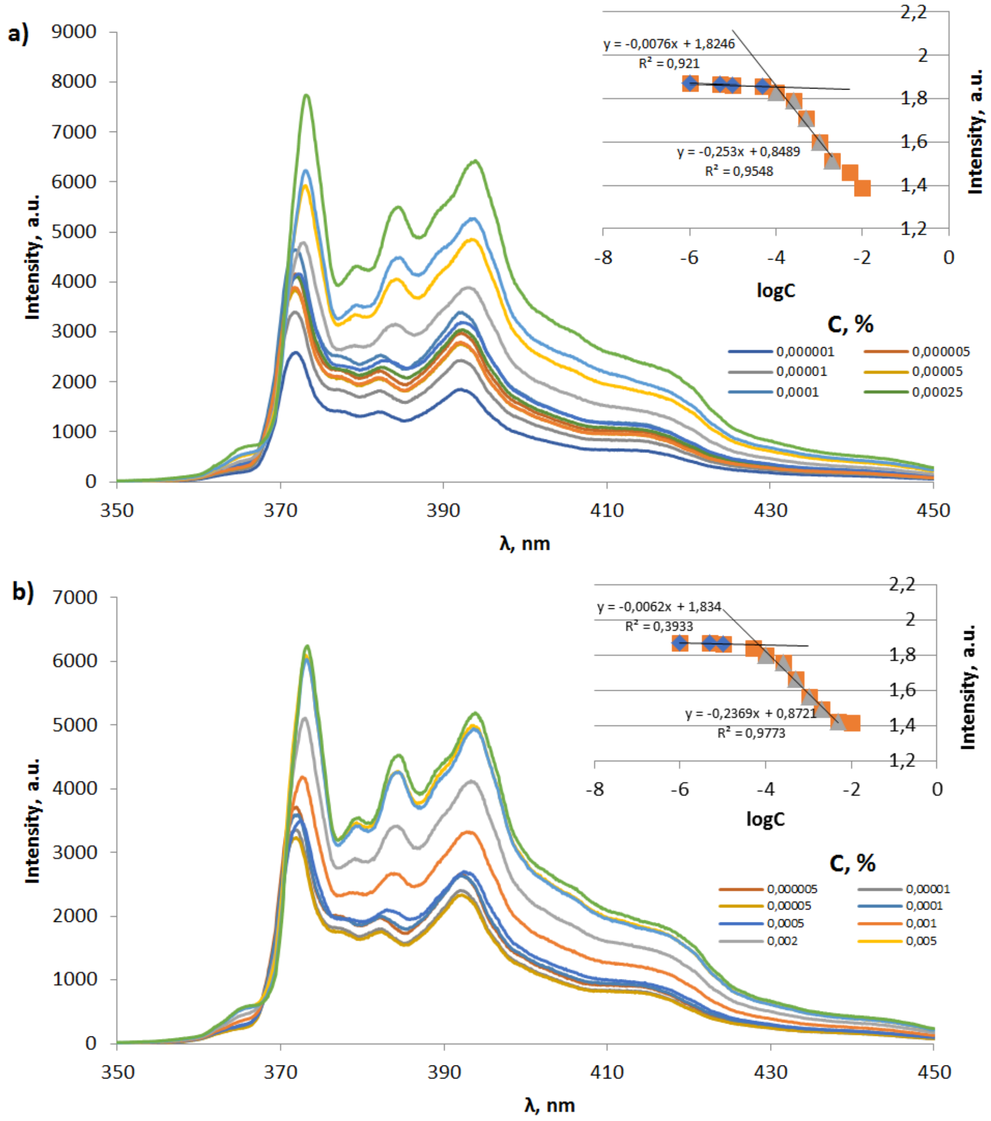

Figure S3: FTIR spectrum of maltodextrin modified using cerium oxide nanoparticles.

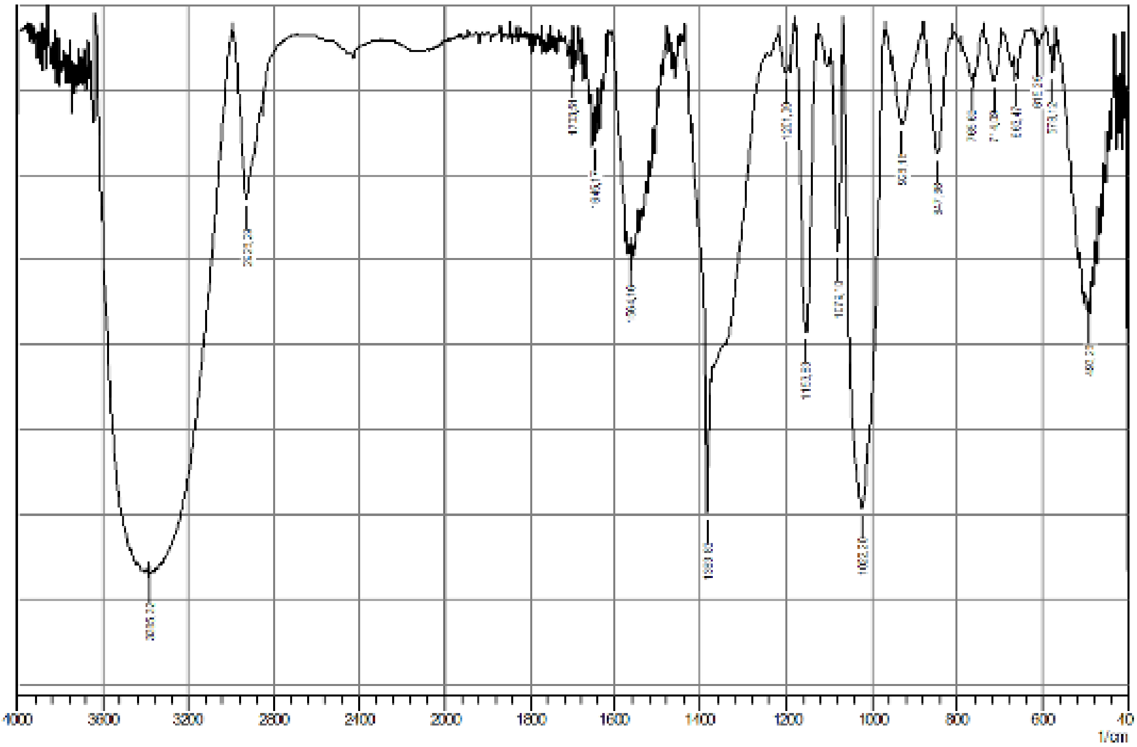

Figure S4: Oxidation of ascorbic acid in an acidic medium using cytochrome *c*.

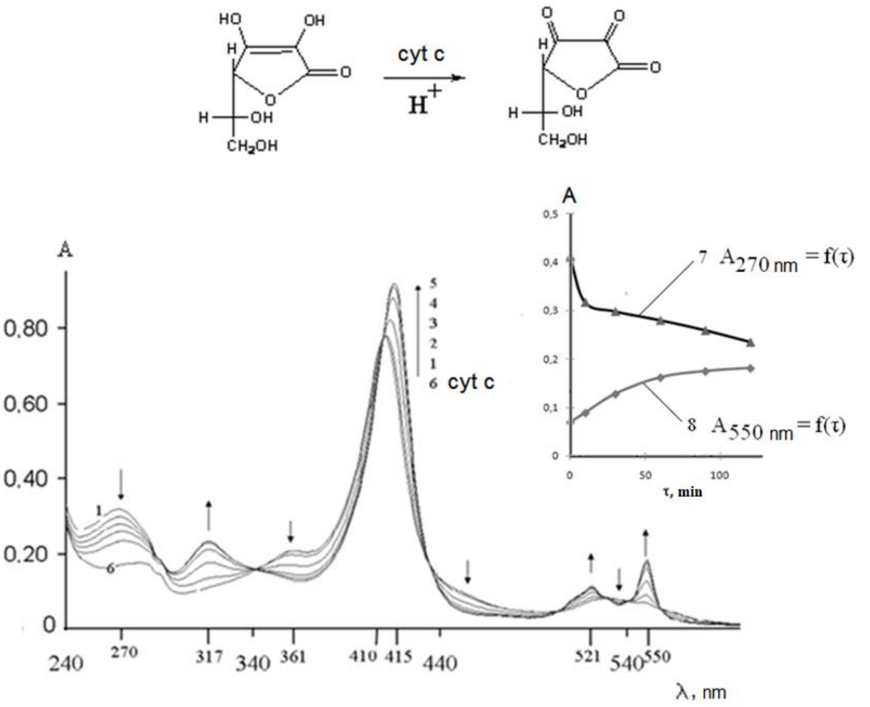

Dynamics of changes in the UV-vis spectra of aqueous solutions of the mixture 6.4·10^{-6} M cyt *c* and 1·10^{-5} M ascorbic acid in time: 1 **– τ=10 min;** 2 **– τ=30 min;** 3 **– τ=60 min;** 4 **– τ=90 min;** 5 **– τ=120 min;** 6 **– 6.4·10^{-6} M cyt *c*; Insert:** 7 **– $A_{270\ nm}$ = f(τ);** 8 **– $A_{550\ nm}$ = f(τ)**

Table S2: Dynamics of cytochrome *c* reduction using ascorbic acid according to electronic absorption spectra.

**Curve (Fig. S3)**	**λ, nm** / **τ, min**	**Absorption, a.u.**						
		270	317	361	410	415	521	550
		Ascorbic acid	Dehydro-ascorbic acid	cyt *c* (ox)	cyt *c* – γ-band (ox)	cyt *c* – γ-band (red)	cyt *c* – β-band (red)	cyt *c* – α-band (red)
-	0	0.4122	0	-	-	-	-	-
1	10	0.3172	0.1367	0.1981	0.7804	-	0.0841	0.0890
2	30	0.2981	0.1757	0.1688	-	0.8128	0.0963	0.1279
3	60	0.2798	0.2115	0.1450	-	0.8814	0.1072	0.1620
4	90	0.2591	0.2282	0.1361	-	0.9083	0.1111	0.1749
5	120	0.2347	0.2328	0.1306	-	0.9209	0.1135	0.1811
6	0	0.1713	0.1128	-	0.7786	-	-	
